# Upadacitinib in Colonic Crohn’s Disease: A Narrative Review of Mechanistic Rationale, Clinical Trial Evidence, and Real-World Outcomes

**DOI:** 10.34172/mejdd.3467

**Published:** 2026-04-30

**Authors:** Yousef VatanParast

**Affiliations:** ^1^Shafa Hospital, Jundishapur University of Medical Sciences, Ahvaz, Iran

**Keywords:** Crohn’s disease, Upadacitinib, JAk inhibitor, Pharmacogenetics, Precision medicine

## Abstract

Crohn’s disease (CD) is a chronic inflammatory bowel disorder with colonic involvement in a substantial proportion of patients, often associated with increased symptom burden and therapeutic complexity. Upadacitinib, a selective Janus kinase 1 (JAK1) inhibitor, has emerged as an effective oral therapy for moderate-to-severe CD, including patients with colonic-predominant disease. This narrative review synthesizes evidence from phase 2 and 3 randomized controlled trials, post-hoc subgroup analyses, long-term extension studies, and real-world observational cohorts evaluating the efficacy and safety of upadacitinib in CD with colonic involvement. Across pivotal trials, induction therapy demonstrated superior rates of clinical remission (approximately 39-50% versus 21-29% with placebo), significant endoscopic response, and rapid symptom improvement. Maintenance therapy sustained both clinical and endoscopic outcomes through one year and beyond. Real-world studies corroborate effectiveness in highly treatment-experienced populations, although heterogeneity in study design limits causal inference. The safety profile is consistent with known JAK inhibitor class effects, with infections—particularly herpes zoster—requiring appropriate risk mitigation. Pharmacogenetic predictors of response to upadacitinib remain undefined, representing an important area for future research. Overall, upadacitinib constitutes a valuable therapeutic option for patients with refractory colonic CD, while comparative effectiveness and precision-medicine strategies warrant further investigation.

## Introduction

 Crohn’s disease (CD) is a heterogeneous inflammatory bowel disease characterized by transmural inflammation that may affect any segment of the gastrointestinal tract. Colonic involvement commonly classified as Montreal L2 disease or colonic-predominant CD is observed in approximately 40-60% of patients and is associated with diarrhea, rectal bleeding, abdominal pain, and an increased risk of complications such as fistulizing disease and hospitalization.^1^

 In this review, the term colonic CD refers to patients with predominant or clinically significant colonic inflammation. It is acknowledged that in pivotal clinical trials, colonic involvement was generally reported as a subgroup characteristic rather than as a prospectively stratified Montreal L2 population, and mixed phenotypes involving both colonic and ileal disease were frequently included.

 Despite major therapeutic advances with biological agents targeting tumor necrosis factor (TNF), integrins, and the interleukin (IL)-12/23 pathway, a substantial proportion of patients experience primary non-response or secondary loss of response.^2^ These limitations have driven interest in orally administered small-molecule therapies that target intracellular inflammatory signaling pathways.

 Upadacitinib is a reversible, selective JAK1 inhibitor that modulates cytokine-mediated signaling via the JAK–signal transducer and activator of transcription (STAT) pathway, which plays a central role in the pathogenesis of CD.^3^ It has received regulatory approval for adults with moderate-to-severe CD who have failed or are intolerant to conventional or biological therapies.^4^ This narrative review critically evaluates the evidence supporting the use of upadacitinib in colonic CD by integrating mechanistic insights, randomized trial data, real-world experience, and long-term outcomes.

## Review Methodology

 This narrative review was informed by a targeted literature search of PubMed and Embase, supplemented by abstracts and presentations from major international gastroenterology congresses (ECCO, DDW, and UEGW). Publications available up to March 2025 were considered. Representative search terms included “upadacitinib,” “Crohn’s disease,” “colonic Crohn’s disease,” “JAK inhibitors,” and “real-world outcomes.”

 Priority was given to phase 2 and 3 randomized controlled trials, long-term extension studies, post-hoc subgroup analyses, and real-world observational cohorts reporting clinical, endoscopic, or safety outcomes. Studies focusing exclusively on pediatric populations, single-patient case reports, and non–peer-reviewed sources were excluded. Particular attention was paid to analyses including patients with colonic involvement; however, most pivotal trials were not prospectively stratified by disease location. As a narrative (non-systematic) review, study selection was not exhaustive, and findings should be interpreted accordingly.

 A schematic flowchart summarizing the literature search strategy and evidence selection process is presented in Figure 1.

**Figure 1:**
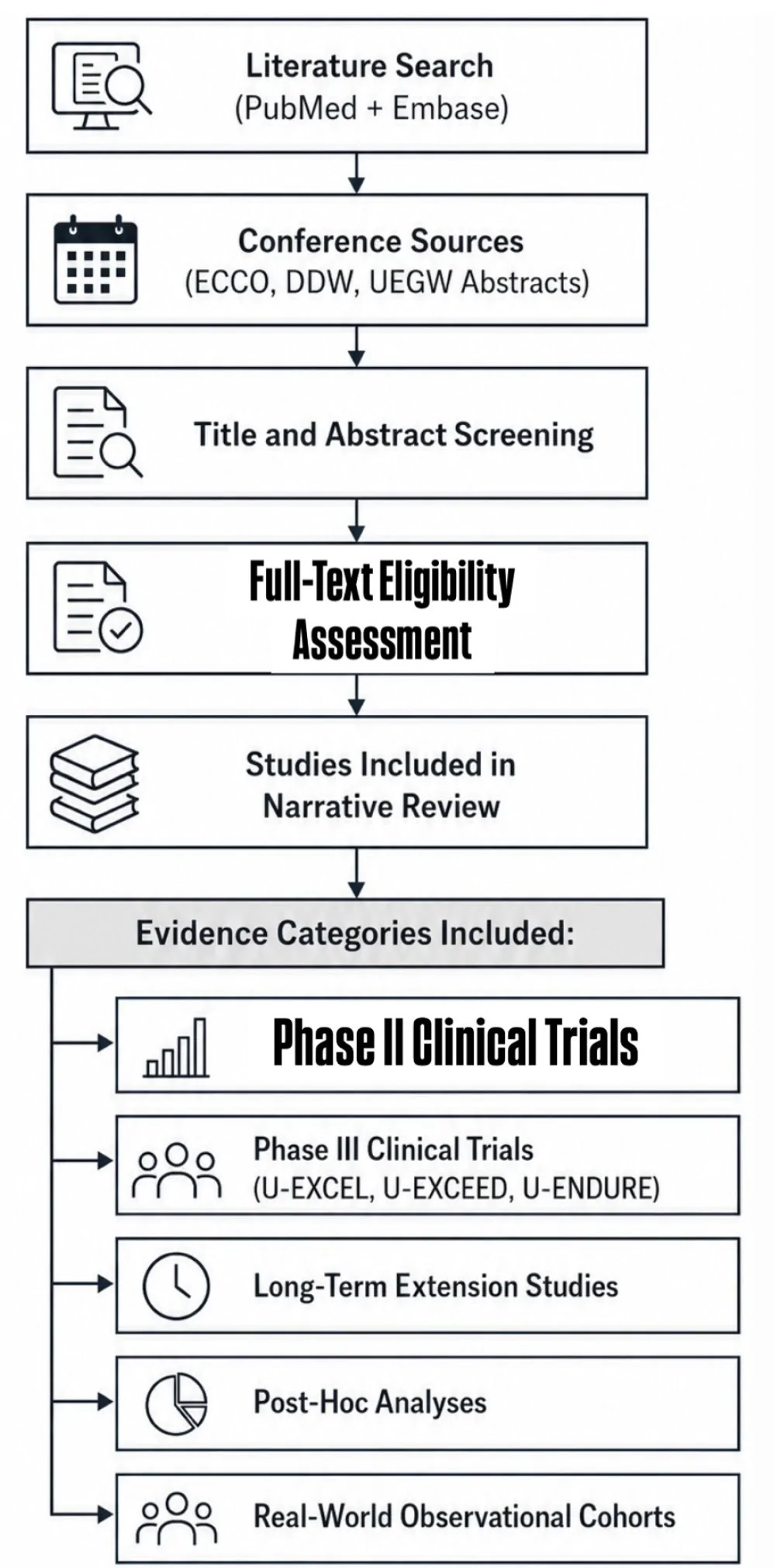
Literature search strategy and evidence selection process for this narrative review

## Mechanism of Action

 Pro-inflammatory cytokines, including IL-6, IL-12, IL-23, and interferon-γ, sustain intestinal inflammation in CD through activation of the JAK–STAT signaling cascade.^5^ Upadacitinib preferentially inhibits JAK1, attenuating downstream STAT phosphorylation while relatively sparing JAK2-dependent pathways involved in hematopoiesis.^6^ Preclinical studies have demonstrated suppression of inflammatory signaling and improvement of experimental colitis following selective JAK1 inhibition ^7^, providing mechanistic support for its therapeutic effects in colonic disease (Figure 2). The pharmacological profile of upadacitinib, including its JAK selectivity, pharmacokinetics, and pharmacodynamic effects on inflammatory biomarkers, is summarized in Figure 3.

**Figure 2:**
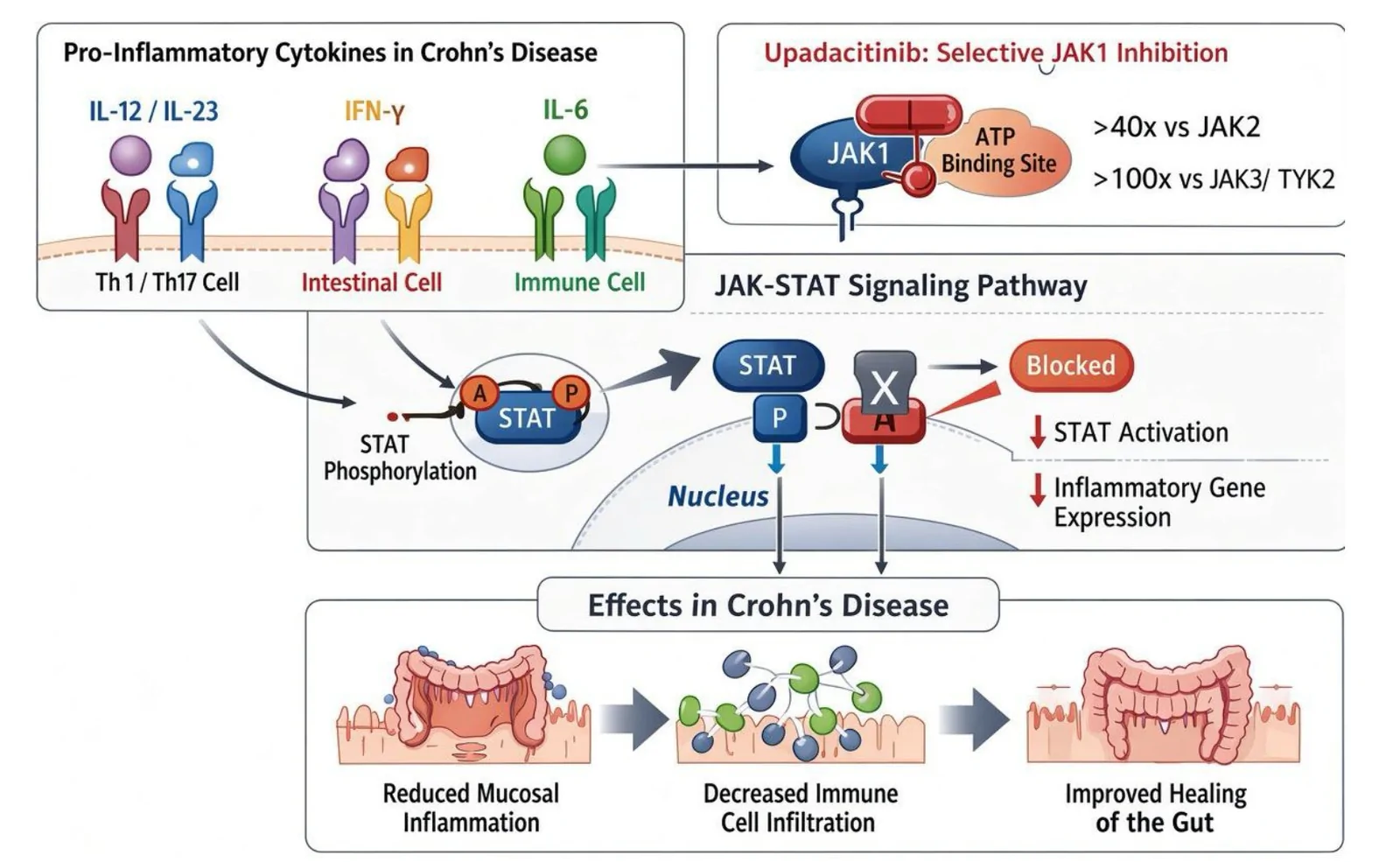
The selective inhibition of JAK1 by upadacitinib and its impact on downstream signaling and intestinal healing are illustrated in the figure

**Figure 3:**
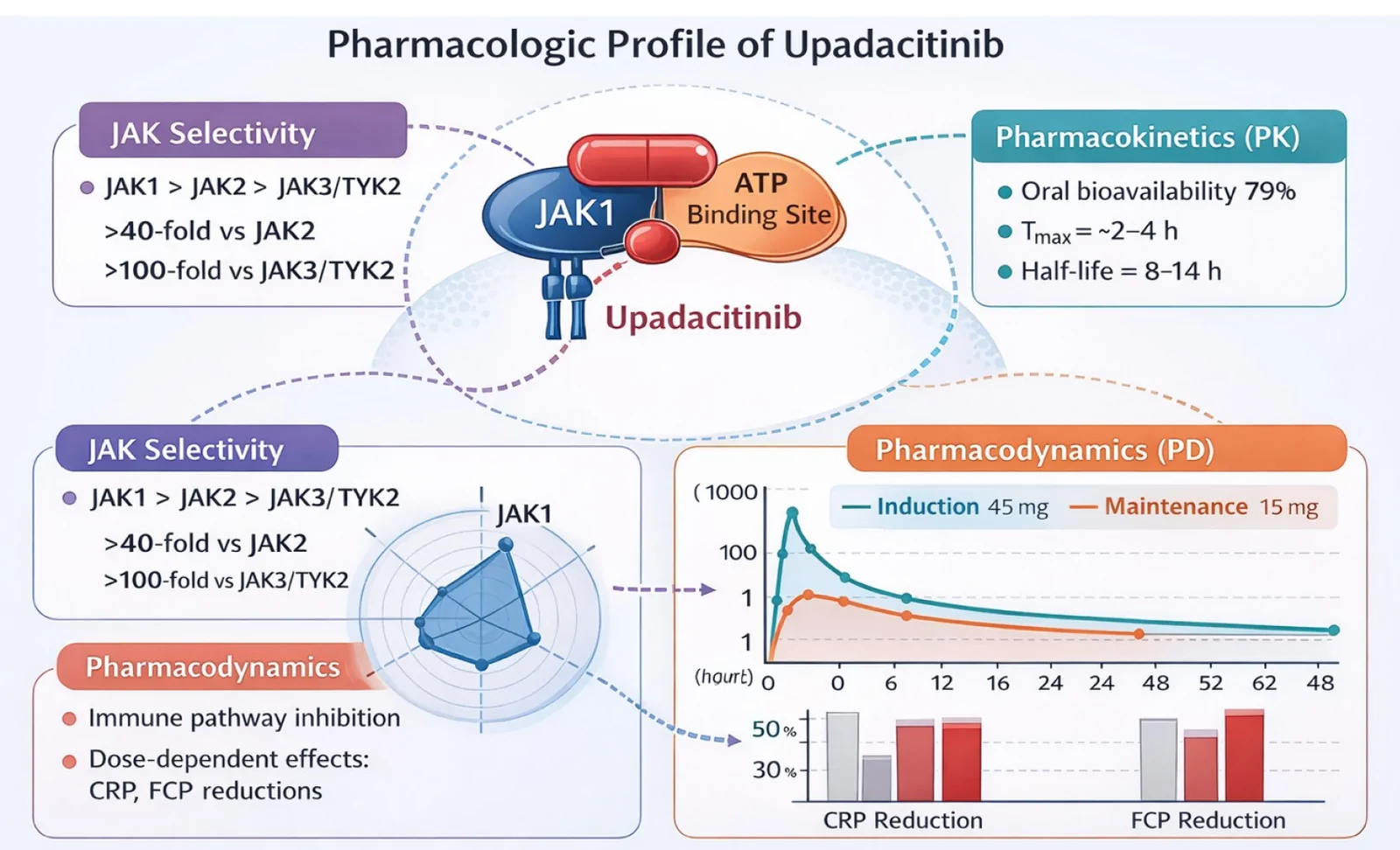
Pharmacological profile of upadacitinib. The diagram illustrates JAK1 selectivity, key pharmacokinetic parameters (oral bioavailability, Tmax, half-life), and pharmacodynamic outcomes, including dose-dependent reductions in CRP and FCP

## Clinical Evidence from Trials

###  Phase 2 Studies

 The CELEST phase 2 trial evaluated the efficacy and safety of upadacitinib in 220 patients with moderate-to-severe CD.^8^ Higher induction doses were associated with dose-dependent improvements in clinical and endoscopic outcomes, with endoscopic remission achieved in up to 22% of treated patients compared with none in the placebo group. Post-hoc subgroup analyses suggested consistent efficacy among patients with colonic involvement, although the study was not powered for disease-location-specific endpoints. In the open-label extension, sustained clinical remission was observed in approximately 30-40% of patients over 30 months ^9^, a finding that should be interpreted with caution given the absence of a comparator arm and potential attrition bias. A summary of the key clinical trials evaluating upadacitinib in CD is presented in Table 1. The overall design and timeline of these pivotal studies are depicted in Figure 4.

**Table 1:** Key clinical trials of upadacitinib in Crohn’s disease

**Study (Phase)**	**Population**	**N**	**Dose**	**Duration**	**Primary Endpoint**	**Main Results**
CELEST (Phase II)	Moderate-to-severe CD, bio-naïve & TNF-IR	220	3, 6, 12, or 24 mg BID	16 weeks	Clinical remission and endoscopic outcomes	Dose-dependent improvements in clinical and endoscopic outcomes; endoscopic remission achieved in up to 22% of treated patients versus 0% with placebo
U-EXCEED (Phase III Induction)	Moderate-to-severe CD, biologic-failure population	495	Upadacitinib 45 mg QD vs placebo	12 weeks	Clinical remission and endoscopic response at Week 12	Clinical remission: 39% vs 21%; Endoscopic response: 35% vs 4%
U-EXCEL (Phase III Induction)	Moderate-to-severe CD, mixed biologic exposure	526	Upadacitinib 45 mg QD vs placebo	12 weeks	Clinical remission and endoscopic response at Week 12	Clinical remission: 50% vs 29%; Endoscopic response: 46% vs 13%
U-ENDURE (Phase III Maintenance)	Patients achieving a clinical response during induction	502	Upadacitinib 15 mg QD, 30 mg QD, or placebo	52 weeks	Maintenance of clinical remission and endoscopic response	Clinical remission was maintained in 47% (15 mg) and 53% (30 mg) versus 20% with placebo; endoscopic response was maintained in 28% and 40% versus 15% with placebo

**Figure 4:**
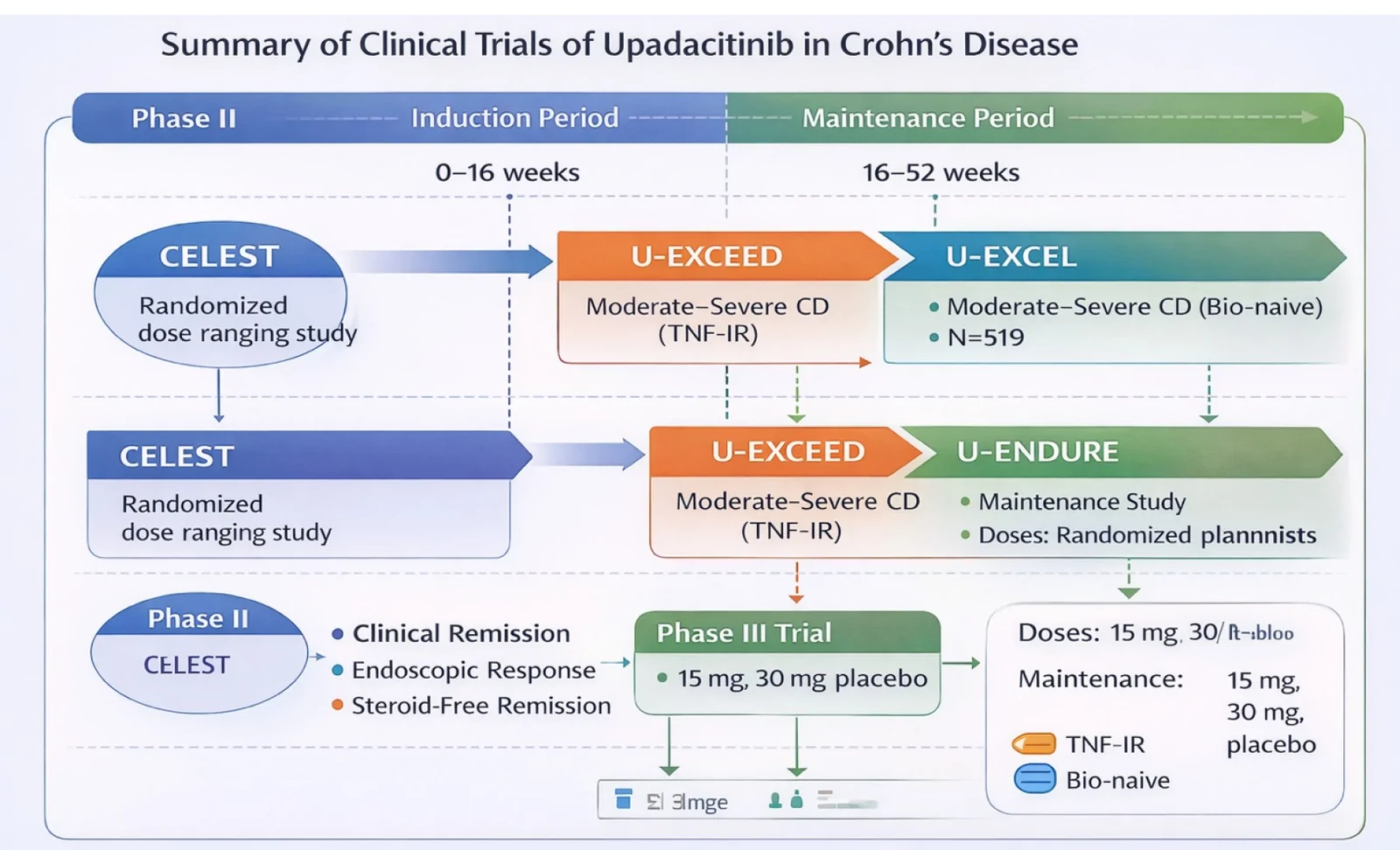
Summary of clinical trials of upadacitinib in Crohn’s disease. The diagram outlines phase 2 (CELEST) and phase 3 (U-EXCEED, U-EXCEL induction, and U-ENDURE maintenance) studies, including patient populations, treatment periods, and key endpoints

###  Phase 3 Induction and Maintenance Trials

 The U-EXCEL and U-EXCEED induction trials and the U-ENDURE maintenance trial enrolled more than 1,000 patients with moderate-to-severe CD.^10^ During induction, upadacitinib 45 mg once daily achieved higher rates of clinical remission (39-50%) compared with placebo (21–29%) and significantly greater endoscopic response (35–46% versus 4–13%). Treatment effects were broadly consistent across predefined subgroups, including patients with reported colonic involvement.

 During maintenance, upadacitinib 15 mg and 30 mg once daily sustained clinical remission in 37-48% and endoscopic response in 28-40% of patients at week 52. Rapid symptom improvement, including reductions in stool frequency and abdominal pain, was observed within the first week of induction therapy.^11^ These efficacy outcomes across induction and maintenance phases are illustrated in Figure 5. Efficacy outcomes in pivotal trials for colonic Crohn’s disease subgroups are presented in Table 2.

**Figure 5:**
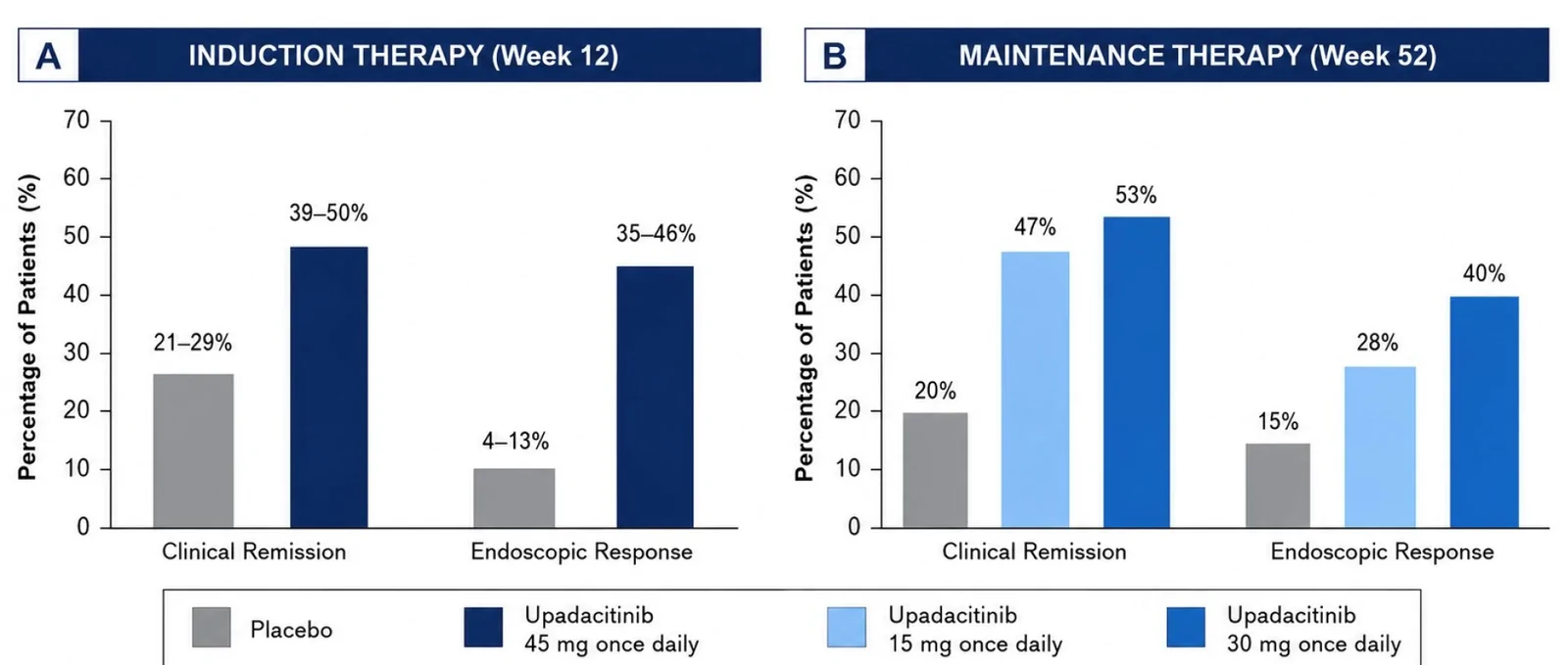
Efficacy outcomes of upadacitinib in colonic CD. Bar charts depict rates of clinical remission, endoscopic response, and biomarker normalization (FCP ≤ 250 μg/g or CRP) in induction (Week 12) and maintenance (Week 52) phases

**Table 2:** Efficacy outcomes in pivotal trials for colonic Crohn’s disease subgroups

**Trial**	**Phase**	**Dose (Induction/Maintenance)**	**Key efficacy endpoints**	**Results in colonic subgroups (upadacitinib vs. placebo)**	**Notes**
U-EXCEED	III (Induction)	45 mg QD / N/A	Clinical remission (week 12); Endoscopic response	Remission: 39% vs. 21%; Endoscopic: 35% vs. 4%	Consistent across colonic involvement; rapid symptom reduction in first week
U-EXCEL	III (Induction)	45 mg QD / N/A	Clinical remission (week 12); Endoscopic response	Remission: 50% vs. 29%; Endoscopic: 46% vs. 13%	Subgroup efficacy similar; includes mixed ileal-colonic phenotypes
U-ENDURE	III (Maintenance)	N/A / 15–30 mg QD	Sustained clinical remission (week 52); Endoscopic response	Remission: 37–48% vs. placebo; Endoscopic: 28–40% vs. placebo	Durable in refractory patients; post-hoc fistula resolution in colonic-associated disease

## Post-Hoc Analyses and Disease-Specific Outcomes

 Post-hoc analyses focusing on perianal fistulizing disease frequently associated with colonic CD—reported higher rates of fistula drainage resolution and closure of external openings with upadacitinib compared with placebo.^12^ These findings should be regarded as hypothesis-generating, given the retrospective design and limited sample sizes.

 Additional analyses demonstrated efficacy irrespective of prior biological exposure, including in patients who had failed multiple advanced therapies.^13^ While supportive of use in refractory disease, these results do not establish comparative superiority over alternative treatment options.

## Long-Term Extension Data

 Two-year results from the U-ENDURE long-term extension study demonstrated durable clinical remission, sustained endoscopic response, improvements in health-related quality of life, and normalization of inflammatory biomarkers among induction responders continuing maintenance therapy.^14^ No new safety signals were identified, supporting the feasibility of longer-term treatment.

## Real-World Evidence

 Real-world observational studies from Europe and North America, including GETAID and UK multicenter cohorts, reported clinical response rates of approximately 60-70% and remission in around half of patients with highly treatment-experienced disease.^15-18^ Many cohorts included patients with colonic-predominant or complex disease phenotypes. However, heterogeneity in patient selection, outcome definitions, and follow-up duration limits direct comparability with randomized trials. Real-world data therefore provide supportive, rather than confirmatory, evidence of effectiveness (Table 3).

**Table 3:** Real-world outcomes in treatment-experienced cohorts

**Cohort/Study**	**Population Characteristics**	**N (Approximate)**	**Key Outcomes**	**Response/Remission Rates**	**Limitations**
GETAID (Europe)	Highly treatment-experienced, colonic-predominant	Not specified	Clinical response/remission	Response: 60–70%; Remission: ~50%	Heterogeneity in design
UK Multicenter	Refractory CD, including complex phenotypes	Not specified	Clinical effectiveness	Similar to GETAID; sustained in follow-up	Observational, no causality
REBOOT IBD (North America)	Bio-failure patients with colonic involvement	Not specified	Symptom improvement, biomarkers	Comparable real-world efficacy	Variable definitions/follow-up

## Comparative Context within Advanced Therapies

 Direct head-to-head trials comparing upadacitinib with other advanced therapies, such as IL-23 inhibitors or sphingosine-1-phosphate receptor modulators, are currently unavailable. In addition, no network meta-analyses have specifically evaluated comparative efficacy within colonic CD subpopulations. While indirect cross-trial comparisons suggest that remission and endoscopic response rates with upadacitinib are numerically comparable to those of other agents particularly in biologic-experienced populations differences in trial design, baseline disease severity, and endpoint definitions preclude definitive conclusions. The oral route of administration and rapid onset of action may represent practical advantages in selected patients.

## Safety Profile

 The safety profile of upadacitinib in CD is consistent with known JAK inhibitor class effects. Infections, including herpes zoster, occur in a dose-dependent manner, underscoring the importance of vaccination and baseline assessment of infection risk prior to therapy initiation. Serious infections were uncommon in clinical trials. Gastrointestinal perforation was rare and typically occurred in patients with additional risk factors, such as concomitant corticosteroid use.

 Regulatory warnings regarding cardiovascular events and malignancy are largely extrapolated from rheumatoid arthritis populations with higher baseline cardiovascular risk. In the CD clinical development program, the incidence of these events was low and comparable to placebo, with no signal amplification observed in long-term extension studies.^10,14^ Careful patient selection remains advisable, particularly in older individuals or those with established cardiovascular risk factors (Table 4). A summary of the safety profile, including adverse events and laboratory abnormalities, is shown in Figure 6.

**Table 4:** Safety profile of upadacitinib in Crohn’s disease trials

**Adverse event category**	**Common events**	**Incidence (upadacitinib vs. placebo)**	**Risk mitigation strategies**	**Long-term observations**
Infections	Herpes zoster, serious infections	Dose-dependent (e.g., herpes zoster: higher with 30 mg); Serious: uncommon	Vaccination, baseline screening	No new signals in 2-year extensions
Gastrointestinal	Perforation	Rare (typically with corticosteroids)	Avoid high-risk combinations	Low incidence, comparable to placebo
Cardiovascular/Malignancy	Events extrapolated from RA data	Low in CD trials; no amplification	Patient selection (avoid high-risk groups)	Comparable to placebo in extensions
Other	Hematologic effects (spared due to JAK1 selectivity)	Minimal impact on hematopoiesis	Monitoring as needed	Consistent with preclinical data

**Figure 6:**
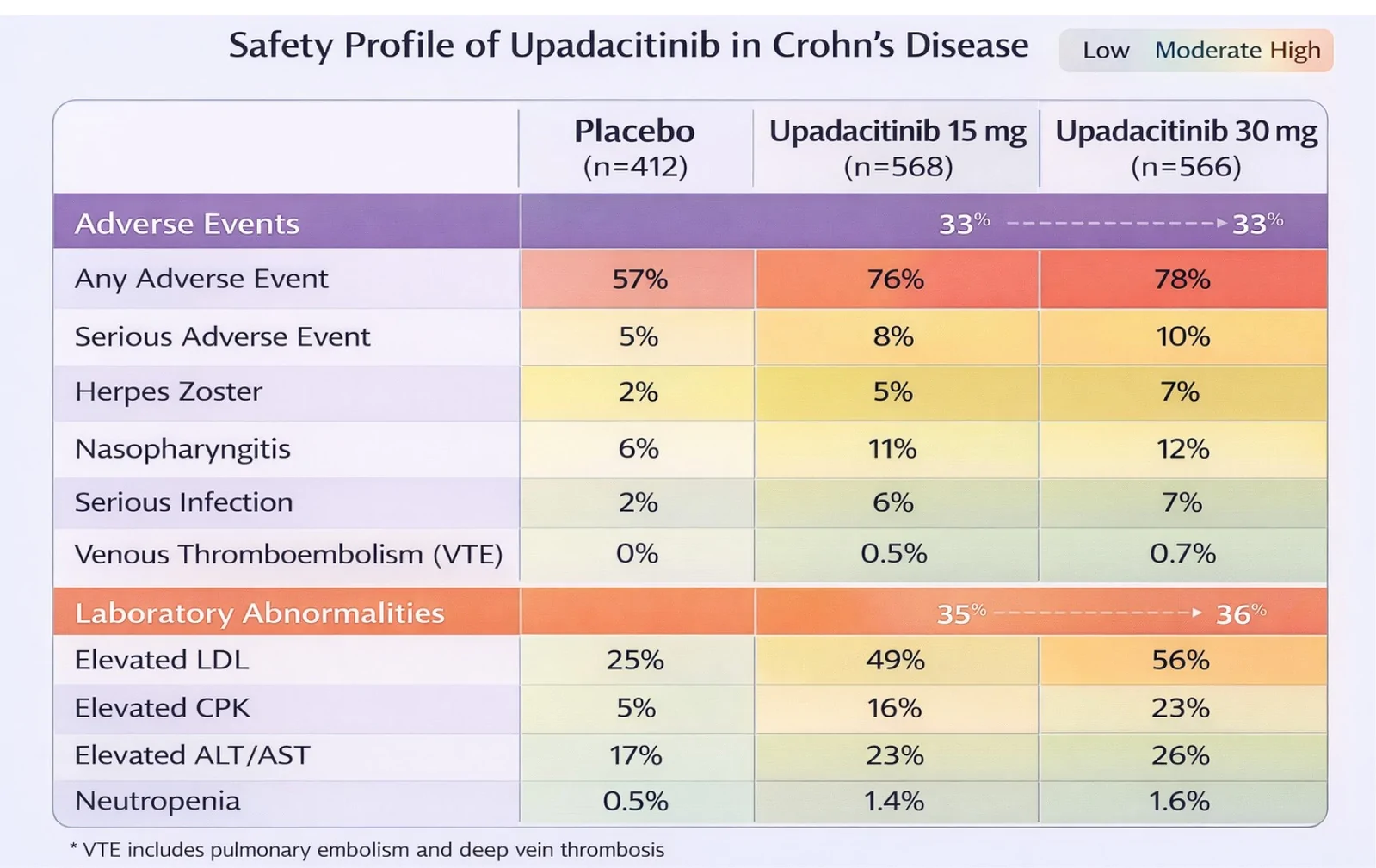
Safety profile of upadacitinib in Crohn’s disease. The table displays incidence rates of adverse events and laboratory abnormalities in the placebo (n = 412), upadacitinib 15 mg (n = 568), and upadacitinib 30 mg (n = 566) groups, with color-coding indicating severity (low to high). VTE includes pulmonary embolism and deep vein thrombosis

## Pharmacogenetic Considerations and Future Directions

 Pharmacogenetics has influenced inflammatory bowel disease management through validated markers for thiopurines and emerging predictors of biological response.^19,20^ For upadacitinib, which is primarily metabolized by CYP3A4, no validated genetic polymorphisms reliably predict treatment response or toxicity. Although variants in JAK–STAT pathway genes contribute to disease susceptibility ^21^, their role in modulating the response to selective JAK1 inhibition remains unproven. Future precision-medicine approaches will likely rely on integrated multi-omics strategies rather than single genetic markers.^22^

## Limitations

 This review is narrative rather than systematic and is therefore subject to selection bias. Long-term extension studies lack comparator arms, and real-world evidence is observational and heterogeneous. Additionally, most clinical trials were not prospectively designed to evaluate colonic-specific outcomes, limiting disease-location-focused conclusions.

## Conclusion

 Upadacitinib has emerged as an effective therapeutic option for patients with moderate-to-severe colonic CD. In pivotal phase 3 studies, induction treatment with upadacitinib 45 mg once daily resulted in rapid clinical response, clinical remission rates of approximately 39-50%, and endoscopic response rates of 35-46%, significantly outperforming placebo. Maintenance therapy with upadacitinib 15 mg or 30 mg once daily sustained these benefits through week 52, with clinical remission achieved in 37-48% of patients and endoscopic response maintained in 28-40%.

 Real-world observational studies have generally confirmed the effectiveness of upadacitinib in highly treatment-experienced populations, including patients with colonic-predominant disease. The safety profile was consistent with the established JAK inhibitor class, with infections, particularly herpes zoster, representing the most frequently reported adverse events, while serious cardiovascular events, malignancies, and gastrointestinal perforation remained uncommon. Appropriate patient selection, vaccination strategies, and ongoing monitoring are important to optimize treatment outcomes. Further comparative and precision-medicine studies are warranted to better define the role of upadacitinib in the management of colonic CD.
